# Poster Session II - A267 SYSTEMATIC REVIEW OF APPENDICEAL CROHN’S DISEASE: DIAGNOSIS AND MANAGEMENT

**DOI:** 10.1093/jcag/gwaf042.267

**Published:** 2026-02-13

**Authors:** I Guo, W Hsu, B Nguyen

**Affiliations:** The University of British Columbia Faculty of Medicine, Vancouver, BC, Canada; The University of British Columbia Faculty of Medicine, Vancouver, BC, Canada; Medicine, The University of British Columbia, Vancouver, BC, Canada

## Abstract

**Background:**

Crohn’s Disease (CD) limited to the appendix is a rare entity that is often not well documented in the literature, and its initial presentation can be difficult to differentiate from acute appendicitis. Diagnosis is often made postoperatively through histopathology. Current knowledge is limited to case reports and small series, standardized guidelines regarding its management.

**Aims:**

We aim to review the current literature surrounding appendiceal Crohn’s Disease, including its clinical presentation, diagnosis, management, and patient outcomes.

**Methods:**

A systematic search of Medline, Embase, Scopus, and PubMed was conducted. Studies reporting primary data on patients whose initial presentation was Crohn’s Disease limited to the appendix were included. Two reviewers (IG, WJH) independently screened and extracted data on demographic characteristics, clinical presentation, initial management, and post-treatment course.

**Results:**

31 studies met inclusion criteria (n = 120 patients). All the studies included are either case reports or retrospective case series. The mean age of diagnosis was 26 years (range 6-68). The mean duration of symptoms prior to presentation was 33 days (range <12 hours to 1 year). Studies that reported the acuity of symptoms instead of specific durations showed 30/39 (77%) acute presentations, 2/39 (5%) subacute, and 7/39 (18%) chronic.

The most common presenting symptom was abdominal pain (95/120, 79%), followed by fever (17/120, 14%), nausea/vomiting (15/120, 13%), diarrhea (9/120, 8%), and anorexia (7/120, 6%). Crohn’s Disease was suspected preoperatively in only 4/120 cases (3%) through imaging/colonoscopy. In contrast, histopathology confirmed CD in 120/120 (100%) of cases based on typical findings including but not limited to transmural inflammation, lymphoid aggregates, and non-caseating granulomas. Appendectomy was performed in 110/120 (92%) of patients.

Median follow up was 3 years (range 1 week to 9 years). 17/110 (15%) cases who underwent appendectomy had recurrence of disease, with 15/17 (88%) of these patients requiring escalation to medical therapy, including steroids, immunomodulators, or biologics. However, long term follow up data was inconsistent across these studies.

**Conclusions:**

Appendiceal Crohn’s Disease is an uncommon entity that is underrecognized in the current literature. While surgical resection of the appendix is often sufficient to manage this entity, the risk of recurrence or progression to ileocecal Crohn’s Disease requiring further management is not uncommon. This systematic review provides the most comprehensive synthesis of the current literature to date.

**Funding Agencies:**

None

